# The Synergistic Effect of Mud Crab Antimicrobial Peptides Sphistin and Sph_12−38_ With Antibiotics Azithromycin and Rifampicin Enhances Bactericidal Activity Against *Pseudomonas Aeruginosa*

**DOI:** 10.3389/fcimb.2020.572849

**Published:** 2020-10-23

**Authors:** Jie Liu, Fangyi Chen, Xiaofei Wang, Hui Peng, Hua Zhang, Ke-Jian Wang

**Affiliations:** ^1^State Key Laboratory of Marine Environmental Science, College of Ocean & Earth Sciences, Xiamen University, Xiamen, China; ^2^State-Province Joint Engineering Laboratory of Marine Bioproducts and Technology, College of Ocean & Earth Sciences, Xiamen University, Xiamen, China

**Keywords:** antimicrobial peptides, Sphistin, Sph_12−38_, *Pseudomonas aeruginosa*, rifampicin, azithromycin, synergistic efficacy

## Abstract

Overuse or abuse of antibiotics has undoubtedly accelerated the increasing prevalence of global antibiotic resistance crisis, and thus, people have been trying to explore approaches to decrease dosage of antibiotics or find new antibacterial agents for many years. Antimicrobial peptides (AMPs) are the ideal candidates that could kill pathogens and multidrug-resistant bacteria either alone or in combination with conventional antibiotics. In the study, the antimicrobial efficacy of mud crab *Scylla paramamosain* AMPs Sphistin and Sph_12−38_ in combination with eight selected antibiotics was evaluated using a clinical pathogen, *Pseudomonas aeruginosa*. It was interesting to note that the *in vitro* combination of rifampicin and azithromycin with Sphistin and Sph_12−38_ showed significant synergistic activity against *P. aeruginosa*. Moreover, an *in vivo* study was carried out using a mouse model challenged with *P. aeruginosa*, and the result showed that the combination of Sph_12−38_ with either rifampicin or azithromycin could significantly promote the healing of wounds and had the healing time shortened to 4–5 days compared with 7–8 days in control. The underlying mechanism might be due to the binding of Sphistin and Sph_12−38_ with *P. aeruginosa* lipopolysaccharides (LPS) and subsequent promotion of the intracellular uptake of rifampicin and azithromycin. Taken together, the significant synergistic antibacterial effect on *P. aeruginosa in vitro* and *in vivo* conferred by the combination of low dose of Sphistin and Sph_12−38_ with low dose of rifampicin and azithromycin would be beneficial for the control of antibiotic resistance and effective treatment of *P. aeruginosa*-infected diseases in the future.

## Introduction

*Pseudomonas aeruginosa* is an opportunistic Gram-negative bacterial pathogen and can cause infections and mass mortality in patients that have cystic fibrosis, AIDS, severe burns, organ transplants, and cancer (Lyczak et al., 2000; Blonder et al., 2004). The current treatment regimen of *P. aeruginosa* includes a wide range of antibiotics including β-lactams, aminoglycosides, fluoroquinolones, or even the inter-combination of those antibiotics (Hancock and Speert, 2000); however, the clinical pathogen *P. aeruginosa* is less susceptible to almost all of the routinely used antibiotics and fairly easy to develop resistance. For example, from 2003 to 2011, the rates of carbapenem-resistant *P. aeruginosa* (CRPA) isolated from patients with hospital-acquired infections in a tertiary care hospital in northeast China were 14.3, 17.1, 21.1, 24.6, 37.0, 48.8, 56.4, 51.2, and 54.1% over time (Xu et al., 2013). In another hospital, First Affiliated Hospital of Nanjing Medical University, in 2008, the resistant rates of *P. aeruginosa* to cephalosporins (Ceftazidime, Cefotaxime, and Cefepime) were 5.9, 82.4, and 17.6%, respectively, while by the end of 2011, only 4 years passed, those numbers increased to 37.8, 85.7, and 27.8%, respectively (Zhang et al., 2015). Owing to its high intrinsic resistance to antibiotics and wide repertoire of virulence factors, the therapy for the *P. aeruginosa*-infected diseases becomes an intractable issue (Hancock and Speert, 2000). As reported, the resistance of *P. aeruginosa* is mainly due to the low permeability of its outer membrane (Hancock, 1998). Besides, the transmembrane efflux pumps are also considered for the intrinsic resistance of *P. aeruginosa* by which the incoming antibiotics can be taken out of the bacteria efficiently (Li et al., 1994). Therefore, exploration of new antipseudomonal agents is desperately needed to take control of the ubiquitous and acute drug resistance of *P. aeruginosa*.

To date, multifarious highlighted new strategies against the multidrug-resistant (MDR) bacteria have been proposed and some potential biological products or pharmaceuticals are expected to be applied in clinic, including antimicrobial peptides (AMPs), anti-virulence compounds, phage therapy, and new molecules (Pacios et al., 2020). For example, both *Enterobacter cloacae* (Mu208) and *Klebsiella pneumoniae* (Mu1343) display multiple heteroresistance to the antibiotics; the simultaneous combinations of antibiotics targeting multiple heteroresistance are effective to kill these two kinds of bacteria, whereas those targeting homogeneous resistance are ineffective (Band et al., 2019). The sequential therapy is considered as a sustainable strategy to counter the antibiotic crisis because this therapeutic method can constrain the emergence of drug resistance and enhance the bactericidal activity (Roemhild and Schulenburg, 2019). Collateral sensitivity means that the mutations in bacteria cause multidrug resistance but simultaneously enhance sensitivity to many other unrelated drugs, and this new mechanism might be developed to alternative antimicrobial strategies against the multidrug bacteria (Pal et al., 2015). AMPs are widespread distributed in various organisms whose many tissues and cell types could produce different functional AMPs (Vizioli and Salzet, 2002; Zasloff, 2002; Brogden et al., 2003). Most AMPs attach to and permeate the target membrane bilayers to induce pore formation and cause the leakage of cytoplasm (Shai, 2002; Brogden, 2005). Besides that, some peptides can alter the septum formation of cytoplasmic membrane and inhibit the synthesis of cell wall, nucleic acid, and protein or enzymatic activity to kill the bacteria (Brogden, 2005). In addition, AMPs can damage the bacterial cell wall, resulting in the radical change of the bacterial morphology, while simple mutations of the bacteria could not reserve the situations (Shai, 2002; Zasloff, 2002; Chongsiriwatana et al., 2008). Although the AMPs possess better antibacterial activity and a broad antibacterial spectrum, antibiotics have not been successfully substituted by AMPs yet. One reason was that the bacteria also developed resistance to AMPs (Habets et al., 2012; Dobson et al., 2013; Makarova et al., 2018; El Shazely et al., 2020); for example, the point mutations induced conformational changes in BraS or BraR, resulting in the constitutive expression of VraDE, conferring *Staphylococcus aureus* to evolve high resistance to nisin A (Arii et al., 2019). The second reason is that there is also cross-resistance of evolved strains to other AMPs, not much but it still exists; for instance, the melittin-resistant *S. aureus* displays cross-resistance against pexiganan (El Shazely et al., 2020). However, despite resistance evolution to AMPs conferred by a few bacteria or cross-resistance of evolved strains to other AMPs, according to the pharmacodynamic studies of AMPs, compared with antibiotics, the evolution of resistance to AMPs is much lower (Yu et al., 2018). Therefore, AMPs are considered to be the potential ideal substituents for antibiotics to be used to some extent in the future. Some studies also have shown that the combination of AMPs with conventional antibiotics has synergistic effect against the targeted pathogenic microorganisms (Li et al., 2017; Zheng et al., 2017; Koppen et al., 2019).

Rifampicin is one kind of derivative of rifamycin. It displays a broad spectrum of antibacterial spectrum against Gram-positive bacteria, particularly *Mycobacteria* and, to a lesser extent, Gram-negative bacteria such as *Escherichia coli, Neisseria meningitides*, etc (Walter and Staehelin, 1971; Heinz Floss and Yu, 2005). The antibacterial mechanism of rifampicin roots in its high affinity binding to and inhibition of the bacterial DNA-dependent RNA polymerase (Campbell et al., 2001). Azithromycin is a kind of macrolide antibiotic, which has a 15-member macrocyclic lactone ring. It is derived from the erythromycin 14-member ring that is inserted into an amino group (Alvarez-Elcoro and Enzler, 1999). Azithromycin also has a broad spectrum of antibacterial spectrum against Gram-positive bacteria including *S. aureus*, parts of *Streptococci, Streptococcus pneumoniae* etc.; Gram-negative bacteria including *Haemophilus influenzae, Haemophilus ducreyi, Neisseria gonorrhoeae, Bordetella pertussis*, etc.; and other pathogens such as *Chlamydia trachomatis, Ureaplasma urealyticum, Mycoplasma pneumoniae*, etc (Retsema et al., 1987; Peters et al., 1992; Alvarez-Elcoro and Enzler, 1999). Azithromycin inhibits the bacterial growth by interfering with their protein synthesis. It could also inhibit RNA-dependent protein synthesis by reversibly binding to the 50 S subunits of the bacterial ribosome (Mazzei et al., 1993; Alvarez-Elcoro and Enzler, 1999).

Our previous studies demonstrate that the AMPs Sphistin (Chen et al., 2015) and Sph_12−38_ (Ma et al., 2017) from the mud crab *Scylla paramamosain* show potent activity against the hospital-acquired opportunistic pathogen *P. aeruginosa* (24 and 12 μmol·L^−1^, respectively). Sphistin is a 38-amino-acid peptide that is derived from the N-terminal of histone H2A in *S. paramamosain*, and Sph_12−38_ is a truncated short fragment of Sphistin. This study aimed to understand whether the clinical medicine azithromycin and rifampicin in combination with Sphistin and Sph_12−38_ would have a synergistic effect on *P. aeruginosa*. *In vitro* experiments were performed using Sphistin in combination with each of two selected antibiotics azithromycin and rifampicin. Furthermore, an *in vivo* study was carried out using a mouse model with wound as infection model and the subsequent treatment was evaluated using Sph_12−38_ in combination with each of azithromycin and rifampicin.

## Materials and Methods

### Peptides, Antibiotics, and Bacterial Strains

The peptides Sphistin (AGGKAGKDSGKSKAKAVSRSARAGLQFPVGRIHRHLK; molecular mass, 3828.48 Da) and Sph_12−38_ (KAKAKAVSRSARAGLQFPVGRIHRHLK; molecular mass, 2983.59 Da) were all synthesized by Shanghai Glory Chemistry Co., Ltd., and the purity of these two peptides reached 98.88% and 98.68%, respectively. The eight medical injections were all purchased from Zhongshan Hospital Xiamen University. The bacterial strain *P. aeruginosa* (ATCC 9027) was purchased from the CGMCC. Bacterial strains were cultivated in Nutrient broth (NB) overnight at 37°C.

### Antimicrobial Activity

After the bacteria were all in logarithmic phase, aliquots of the bacterial cell suspension (~5 × 10^5^ CFU·ml^−1^) were then added to 96-well plates; each well-contained 100 μl of cell suspension. The peptides and antibiotics were all dissolved in sterile water, and the final concentration of the antibacterial agents ranged from 1.5 to 48 μmol·L^−1^, and then twofold serial dilutions of the peptide and antibiotics were mixed with the bacteria with an equal volume. The samples were subsequently incubated at 37°C for 24 h. The minimal inhibitory concentrations (MICs) were defined as the lowest concentration of antibacterial agents that completely inhibited bacterial growth. The MICs of the antibacterial agents against the tested microorganisms were determined by the standard broth microdilution method (Khara et al., 2014; Yamamoto and Tamura, 2014).

### Synergistic Effect Assay

The synergistic effects of Sphistin and Sph_12−38_ in combination with the antibiotics were tested using the checkerboard assay as previous research described (Rand et al., 1993; Petersen et al., 2006). Twofold serial dilutions of Sphistin, Sph_12−38_, and the antibiotics were prepared, the peptides were mixed in a 1:1 volume ratio with the antibiotics, and then the mixture (100 μl) was added into 96-well plates. The equal volume of bacterial suspension (~5 × 10^5^ CFU·ml^−1^) was seeded into the plates and incubated with the antibacterial agent mixture at 37°C for 24 h. To ensure the precision of experimental results, each assay was in triplicate and repeated three times. The fractional inhibitory concentration index (FICI) was used to assess the synergistic effects of the combination of AMPs with antibiotics. The FICI could be calculated by the formula: FICI = [MIC_AMPs_ in synergistic system]/[MIC_AMPs_ alone] + [MIC_Antibiotics_ in synergistic system]/[MIC_Antibiotics_ alone] (Pankey and Ashcraft, 2005; Pankey et al., 2005). When FICI <0.5, it was interpreted as synergy; 0.5 ≤ FICI < 1.0, partial synergy; 1.0 ≤ FICI < 4.0, additive effect; and FICI ≥ 4.0, antagonism (Odds, 2003).

### The Time-Course Killing Kinetics

The time-course killing kinetics were assayed using *P. aeruginosa* (ATCC 9027) in the presence of Sphistin (6 μmol·L^−1^ 1/4 × MIC), azithromycin (18 μg·ml^−1^ 1/10 × MIC), and a combination of Sphistin (6 μmol·L^−1^ 1/4 × MIC) with azithromycin (18 μg·ml^−1^ 1/10 × MIC); for Sphistin and/or rifampicin, they are as follows: Sphistin (1.5 μmol·L^−1^ 1/16 × MIC), rifampicin (0.625 μg·ml^−1^ 1/4 × MIC), and a combination of Sphistin (1.5 μmol·L^−1^ 1/16 × MIC) with rifampicin (0.625 μg·ml^−1^ 1/4 × MIC). The bacterial cells were cultured overnight and further cultured in new medium, the next day to reach the logarithmic phase, and then incubated with Sphistin and/or rifampicin for an additional 0, 5, 15, 30, 45, 60, and 120 min at 37°C; meanwhile, the experimental bacteria were also incubated with Sphistin and/or azithromycin for an additional 0, 30, 60, 90, 120, 240, and 360 min at 37°C. The total treated bacterial population was then plated on NB agar plates and continued to be incubated overnight at 37°C, and finally we counted the colonies.

### Live/Dead Assay

The *P. aeruginosa* strain (ATCC 9027) was cultured at 37°C until the bacterial cells reached the logarithmic phase, and then the bacterial cells were harvested and washed twice using the NB. The pellet was resuspended to ~10^6^ CFU·ml^−1^ in the same buffer, after which the prepared bacteria were treated as mentioned above. After the bacteria were treated with all the antibacterial agents, all the bacteria were harvested and stained with SYTO 9 and propidium iodide (PI) in the ratio of 1:1 from the LIVE/DEAD® *Bac*Light^TM^ Bacterial Viability Kits (Thermo Fisher Scientific). After mixing all the mixture thoroughly, the CytoFLEX flow cytometry (Beckman Coulter, California, USA) was used to test the cell membrane integrity and cell viability of the bacteria.

### Scanning Electron Microscopy

*P. aeruginosa* (ATCC 9027) cells in mid-log phase were suspended in PBS to ~1 × 10^7^ CFU·ml^−1^, after which aliquots were treated as mentioned above. The control group was treated with DPBS (2.45 g Na_2_HPO_4_·12H_2_O and 0.49 g NaH_2_PO_4_·2H_2_O dissolved in 1,000 ml of sterile water, pH = 7.4). After incubation, the bacterial cell pellets were harvested and fixed in 2.5% glutaraldehyde for 2 h at 4°C, followed by two washes in DPBS. The fixed cells were dehydrated for 15 min using a graded ethanol series (30, 50, 70, 90, and 100%). Then, the cells were dehydrated for 5 min in tertiary butanol, this operation was repeated 10 times, and finally, the samples were immersed in tertiary butanol overnight at 4°C. When the prepared specimens dried, conductive coating was applied to the specimens and they were examined using a field emission scanning electron microscopy (SUPRA 55; ZEISS, Germany).

### Bacterial Cell Membrane Permeabilization Assay

The permeability of bacterial cell membranes was determined by measuring the leakage of intracellular ATP levels out of the bacterial cells as described by previous research (Koshlukova et al., 1999). Briefly, *P. aeruginosa* (ATCC 9027) was cultured overnight at 37°C, the cells were harvested and washed twice, and the bacterial cells were resuspended in DPBS. The prepared bacterial cells were treated as mentioned above. After incubation, samples were centrifuged to get the supernatant and then 10 μl of the supernatant was added into 90 μl of the standard reaction solution that comes from the Molecular Probes' ATP Determination Kit (Thermo Fisher Scientific). Prior to testing the luminescence of the samples, use the luminometer to measure the background luminescence and then subtract the background luminescence and read the luminescence of the samples. Using the gradient dilution ATP standard solution to generate a standard curve for a series of ATP concentrations, and according to the standard curve, we could calculate the amounts of the leakage of the intracellular ATP.

### Transmission Electron Microscopy (TEM)

The treated bacteria were fixed in 2.5% glutaraldehyde overnight at 4°C, the samples were washed in PBS, the bacteria were harvested and resuspended in PBS (1.5 × 10^9^ CFU·ml^−1^), and the samples were added into the agar models. The mixture was centrifuged and the supernatant was removed, and the prepared samples were put into 2% molten agar solution, after which the mixed soulution was served on ice until agar solution solidification. The agar block was cut into the size of a rice grain and washed twice, and the agar granules were resuspended and fixed in 2.5% glutaraldehyde overnight at 4°C. Finally, the fixed agar granules were suspended in PBS; after embedding and sectioning, the samples were examined by TEM (Tecnai G2 Spirit, FEI, USA).

### Closure of Wounds Infected With *P. aeruginosa*

Six to eight week old BALB/c male mice weighting 25–28 g (*n* = 42) were used in the study. The wound was produced by using the medical pressure-sensitive adhesive tape to remove a 2 cm × 2 cm area of the epidermis on the backs of mice. *P. aeruginosa* (ATCC 9027) (1 × 10^8^ CFU per 20 μl in PBS) was then immediately smeared onto the artificial wound. Two hours after bacterial infection at the wound site, rifampicin (1.25 μg·ml^−1^, 1/2 × MIC), azithromycin (90 μg·ml^−1^, 1/2 × MIC), Sph_12−38_ (24 μmol·L^−1^, 2 × MIC) alone and in combination with rifampicin (1.25 μg·ml^−1^, 1/2 × MIC), and azithromycin (90 μg·ml^−1^, 1/2 × MIC), respectively, in 20 μl of PBS were administered in the wound site by hypodermic injection. The mice without infection of *P. aeruginosa* (ATCC 9027) were used as uninfected controls. The wounds were photographed at a definite time to record the wound healing.

### Statistical Analysis

All experiments were performed three independent times, with each sample performed in triplicate. All data were expressed as means ± standard deviations. Differences among groups were evaluated by using one-way analysis of variance. *P* < 0.05 were considered statistically significant.

## Results

### The Synergistic and Additive Antibacterial Effects of Sphistin and Sph_12−38_ in Combination With Eight Commonly Used Antibiotics

As reported previously (Chen et al., 2015), the synthetic Sphistin has no cytotoxicity toward mouse osteoblastic cell MC3T3-E1 and crab hemocytes even at high tested concentrations (100 mg·ml^−1^). Similarly, Sph_12−38_ also exhibits no cytotoxicity on HeLa cell and crab hemocytes (Ma et al., 2017). Both of the AMPs had strong antibacterial activity and also showed potent activity against *P. aeruginosa*, whose MIC values were 24 and 12 μmol·L^−1^, respectively. The antimicrobial activities of Sphistin and Sph_12−38_ in combination with the commonly used clinical antibiotics rifampicin, vancomycin, penicillin, ceftizoxime, cefotiam, clindamycin, tinidazole, and azithromycin against *P. aeruginosa* were individually determined using the broth microdilution method in accordance with the Clinical and Laboratory Standards Institute (CLSI) recommendation (C. L. S. Institute, 2012), and the results are summarized in Tables 1, 2. Among the eight selected antibiotics, only Sphistin and Sph_12−38_ in combination with azithromycin and rifampicin exhibited significant synergistic activity against *P. aeruginosa*. The mixture of 4-fold reduction of Sphistin (reduced from 24 to 6 μmol·L^−1^) with 10-fold reduction of azithromycin (reduced from 180 to 18 μg·ml^−1^) and the mixture of 16-fold reduction of Sphistin (reduced from 24 to 1.5 μmol·L^−1^) with 4-fold reduction of rifampicin (reduced from 2.5 to 0.625 μg·ml^−1^) could inhibit the growth of *P. aeruginosa*. Similar situations also occurred when Sph_12−38_ in combination with azithromycin and rifampicin and the mixture of 8-fold reduction of Sph_12−38_ (reduced from 24 to 1.5 μmol·L^−1^) with 10-fold reduction of azithromycin (reduced from 180 to 18 μg·ml^−1^) or with 4-fold reduction of rifampicin (reduced from 2.5 to 0.625 μg·ml^−1^) could also significantly minimize the growth of *P. aeruginosa*. The FICIs of these four combinations were all <0.5 (Tables 1, 2), which could be considered as a synergistic effect. However, for other antibiotics, including vancomycin, penicillin, ceftizoxime, cefotiam, clindamycin, and tinidazole, when they were combined with either Sphistin or Sph_12−38_ to treat *P. aeruginosa*, the FICIs were all more than 1, which showed no synergistic effect.

**Table 1:** MIC data for Sphistin alone and in combination with antibiotics against *P. aeruginosa*.

**Compounds**	**MIC Antibiotics (μg·ml^**−1**^)**	**FIC Antibiotics**	**MIC Sphistin (μmol·L^**−1**^)**	**FIC Sphistin**	**FIC index**
Vancomycin	4.5	>2.25	24	>12	>1
Penicillin	>18	>9	24	>12	>1
Ceftizoxime	>20	>10	24	>12	>1
Cefotiam	>30	>15	24	>12	>1
Clindamycin	>25	>12.5	24	>12	>1
Tinidazole	>12	>6	24	>12	>1
Azithromycin	180	18	24	6	0.35
Rifampicin	2.5	0.625	24	1.5	0.3125

**Table 2:** MIC data for Sph_12−38_ alone and in combination with antibiotics against *P. aeruginosa*.

**Compounds**	**MIC Antibiotics (μg·ml^**−1**^)**	**FIC Antibiotics**	**MIC Sph_**12−38**_ (μmol·L^**−1**^)**	**FIC Sph_**12−38**_**	**FIC index**
Vancomycin	4.5	>2.25	12	>6	>1
Penicillin	>18	>9	12	>6	>1
Ceftizoxime	>20	>10	12	>6	>1
Cefotiam	>30	>15	12	>6	>1
Clindamycin	>25	>12.5	12	>6	>1
Tinidazole	>12	>6	12	>6	>1
Azithromycin	180	18	12	1.5	0.225
Rifampicin	2.5	0.625	12	1.5	0.375

### Effects of Sphistin With Azithromycin and Rifampicin on Viability of *P. aeruginosa*

**Figure 1:**
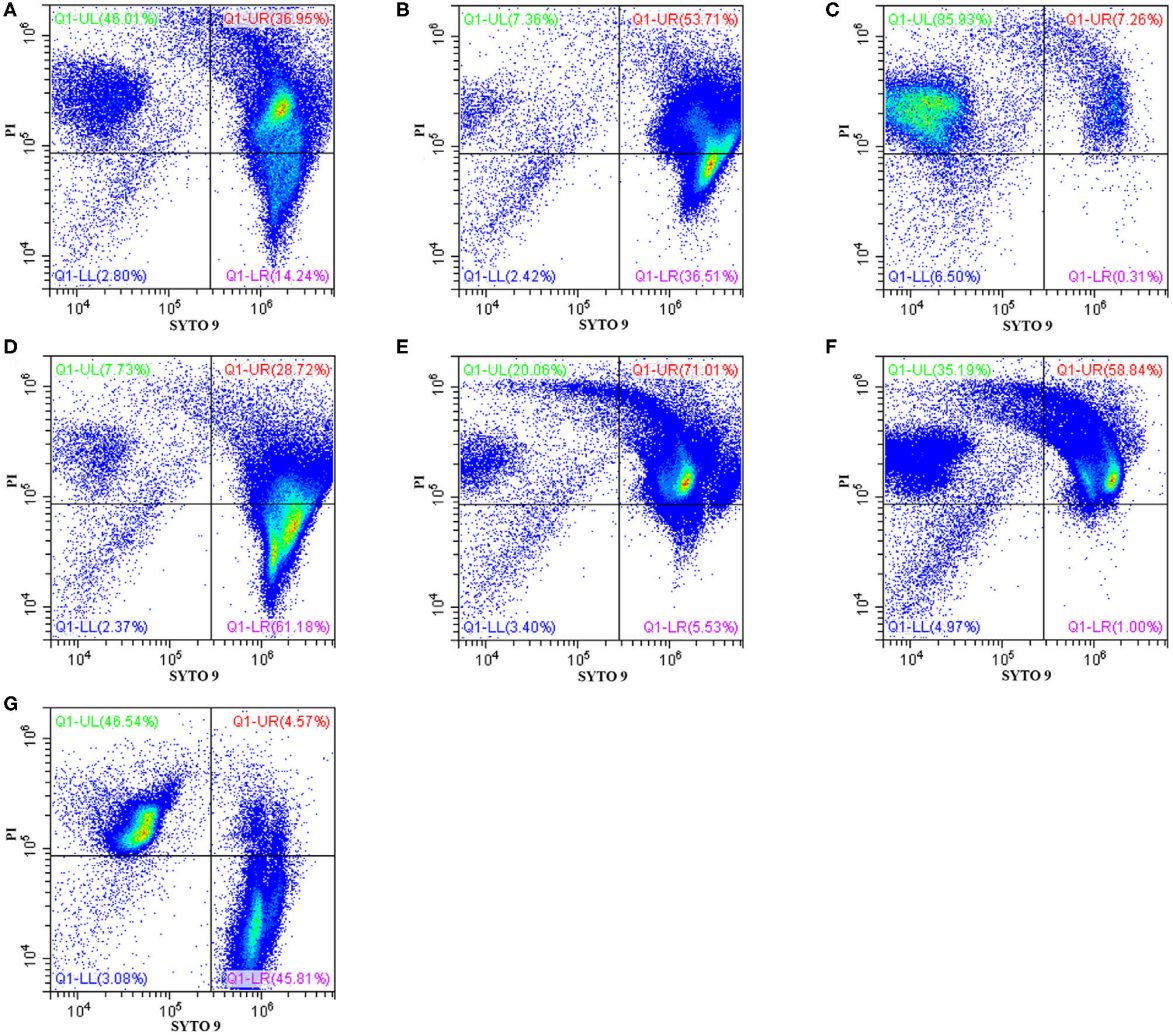
Flow cytometry showing *P. aeruginosa* exposed to Sphistin, azithromycin, rifampicin, Sphistin in combination with azithromycin, and Sphistin in combination with rifampicin. The bacteria were all incubated with **(A)** 6 μmol L^−1^ Sphistin, **(B)** 18 μg·ml^−1^ azithromycin, **(C)** a combination of 6 μmol·L^−1^ Sphistin and 18 μg·ml^−1^ azithromycin for 4 h at 37°C; and **(D)** 1.5 μmol·L^−1^ Sphistin, **(E)** 0.625 μg·ml^−1^ rifampicin, **(F)** a combination of 1.5 μmol·L^−1^ Sphistin and 0.625 μg·ml^−1^ rifampicin for 2 h at 37°C. Then, the bacteria were all stained with SYTO 9 and propidium iodide (PI) fluorescent nucleic acid stain. **(G)** The same amounts of living bacteria and completely dead bacteria were stained with SYTO 9 and PI, respectively. Bacteria were stained with SYTO 9, which showed living cells in the Q1-LR quadrant, while those bacterial cells that were only stained with PI were all in the Q1-UL quadrant; in the Q1-UR quadrant, the bacterial cells were stained by the SYTO 9 and PI together. The results were detected by flow cytometry.

The effects of Sphistin in combination with azithromycin and rifampicin on viability and membrane integrity of *P. aeruginosa* were tested by using the LIVE/DEAD® *Bac*Light^TM^ Bacterial Viability Kits and flow cytometry. This kit has two-color fluorescence: the SYTO 9 green-fluorescent nucleic acid stain, which could stain all living cells green, and the red-fluorescent nucleic acid stain, PI, which could specifically penetrate the bacterial cells such that the cell membrane is damaged and cells are stained red. When the flow cytometry was used to detect the mixture of the same number of living bacteria and completely dead bacteria, the living bacteria were stained with SYTO 9, and they were all almost distributed in the Q1-LR quadrant, while the completely dead bacteria were stained with PI, and they were all almost distributed in the Q1-UL quadrant (Figure 1G). The results showed that ~46.01% and ~7.36% of the bacteria treated with Sphistin and azithromycin, respectively, were stained by PI (Figures 1A,B), and the combination of Sphistin and azithromycin could totally kill 85.93% of the bacterial cells (Figure 1C). As for Sphistin and/or rifampicin treatment, only ~7.73% and ~20.06% of the bacterial cells were completely killed by Sphistin and rifampicin, respectively (Figures 1D,E), while when Sphistin is in combination with rifampicin, ~35.19% of the bacterial cells were completely killed (Figure 1F). These findings indicated that exposure to both Sphistin in combination with azithromycin or rifampicin resulted in the uptake of PI by more bacterial cells than Sphistin and these two antibiotics alone, suggesting a significant increase in cell permeability and hence the synergistic activity of Sphistin in combination with these two antibiotics, especially with the azithromycin.

### The Time-Course Killing Kinetics

**Figure 2:**
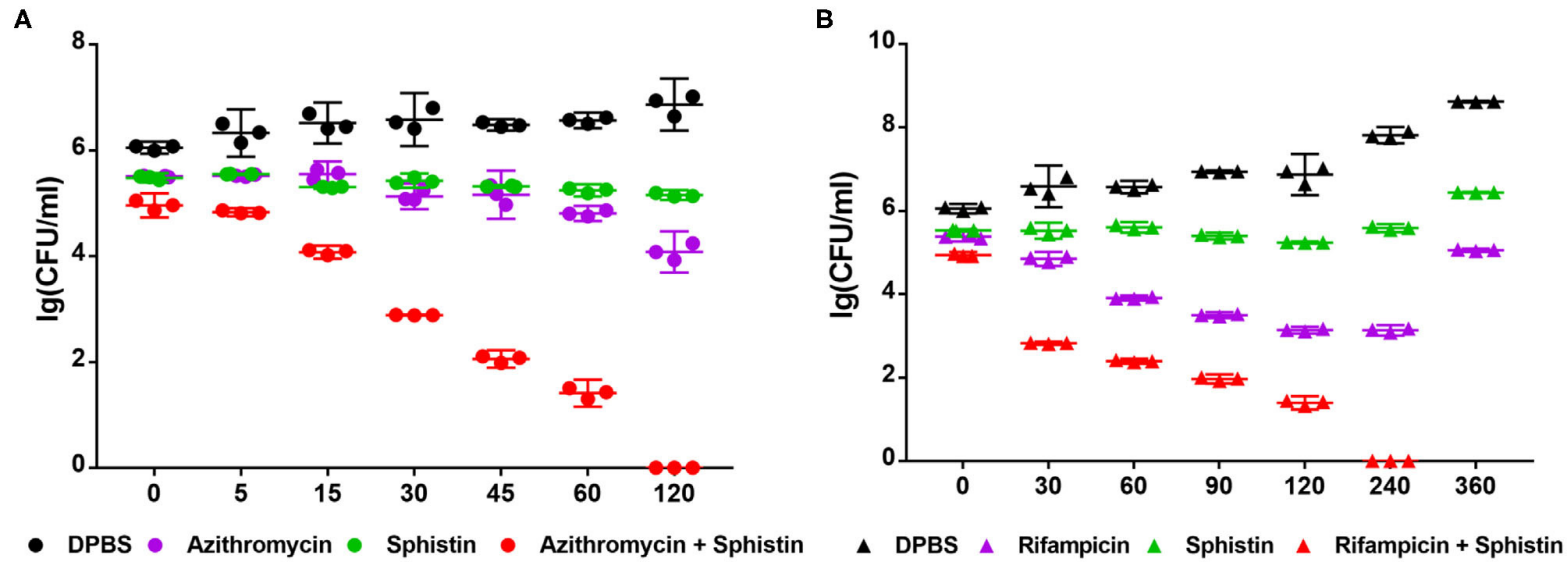
Growth curves of *P. aeruginosa* when incubated with **(A)** DPBS, Sphistin (6 μmol·L^−1^), azithromycin (18 μg·ml^−1^), and Sphistin (6 μmol·L^−1^) in combination with azithromycin (18 μg·ml^−1^) for 2 h; **(B)** DPBS, Sphistin (1.5 μmol·L^−1^), rifampicin (0.625 μg·ml^−1^), and Sphistin (1.5 μmol·L^−1^) in combination with rifampicin (0.625 μg·ml^−1^) for 6 h.

According to the results, Sphistin in combination with azithromycin and rifampicin had synergistic effects against *P. aeruginosa*. We also conducted a time-course killing experiment to examine the effects of Sphistin and/or these two antibiotics against *P. aeruginosa*. Sphistin in combination with azithromycin reduced the number of bacteria by more than two orders of magnitude after 45 min, and after 2 h, all the bacteria were killed. By contrast, the Sphistin or azithromycin used alone did not inhibit the bacterial viability efficiently (Figure 2A). Similarly, Sphistin in combination with rifampicin also inhibited the growth of *P. aeruginosa*; after 1 h, the number of bacteria was also reduced more than two orders of magnitude, and the combination of Sphistin and rifampicin could kill all the bacteria after 4 h. However, if Sphistin or rifampicin was incubated with the bacteria alone, each of them could not inhibit bacterial viability, and the concentration of bacteria increased after 4 h (Figure 2B).

### Visualization of the Interaction of Sphistin and/or Rifampicin and Azithromycin With *P. aeruginosa*

**Figure 3:**
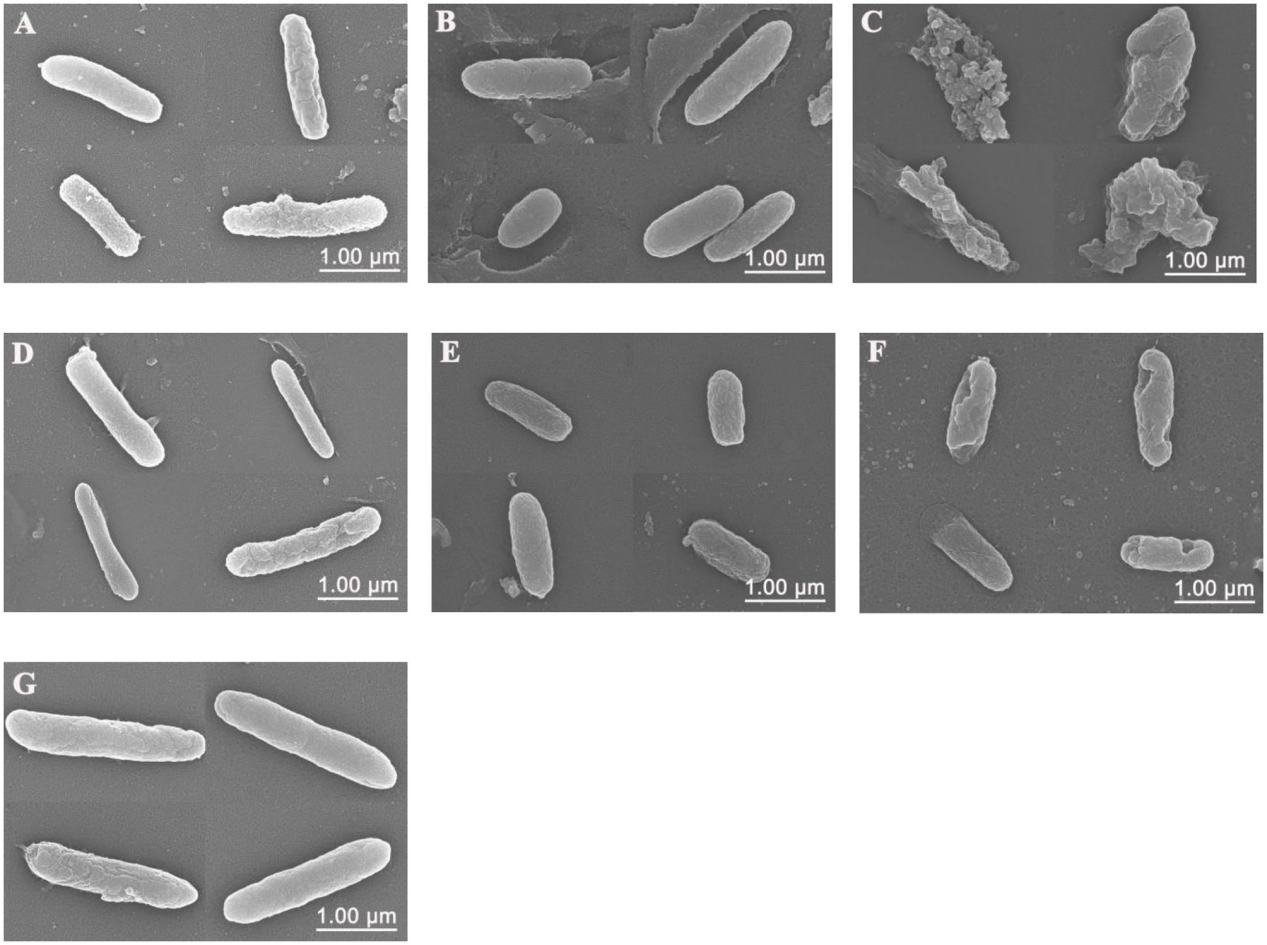
Scanning electron microscope (SEM) images of *P. aeruginosa* treated with **(A)** 6 μmol·L^−1^ Sphistin, **(B)** 18 μg·ml^−1^ azithromycin, and **(C)** a combination of 6 μmol·L^−1^ Sphistin and 18 μg·ml^−1^ azithromycin for 2 h at 37°C. Meanwhile, the bacteria treated with **(D)** 1.5 μmol·L^−1^ Sphistin, **(E)** 0.625 μg·ml^−1^ rifampicin, **(F)** a combination of 1.5 μmol·L^−1^ Sphistin and 0.625 μg·ml^−1^ rifampicin, and **(G)** DPBS for 4 h at 37°C.

Scanning electron microscopy (SEM) was used to visualize the bacterial cell membrane damaged by Sphistin and/or rifampicin and azithromycin. Compared with the control group (Figure 3G), *P. aeruginosa* treated with Sphistin or azithromycin alone showed slight cell shrinkage (Figures 3A,B), but the cell membrane was intact. When the bacteria were incubated with a combination of Sphistin and azithromycin, the entire cell membrane was completely damaged along with the leakage of cytoplasmic contents (Figure 3C). When Sphistin or rifampicin was used alone, each reagent only induced slight changes in cellular morphology (Figures 3D,E); however, when the bacteria were treated with Sphistin in combination with rifampicin, obvious depressions were observed on the bacterial cell membrane, but no leakage of cytoplasmic content was present and the cellular morphology remained intact (Figure 3F).

### Antimicrobial Mechanism of Sphistin in Combination With Rifampicin and Azithromycin

**Figure 4:**
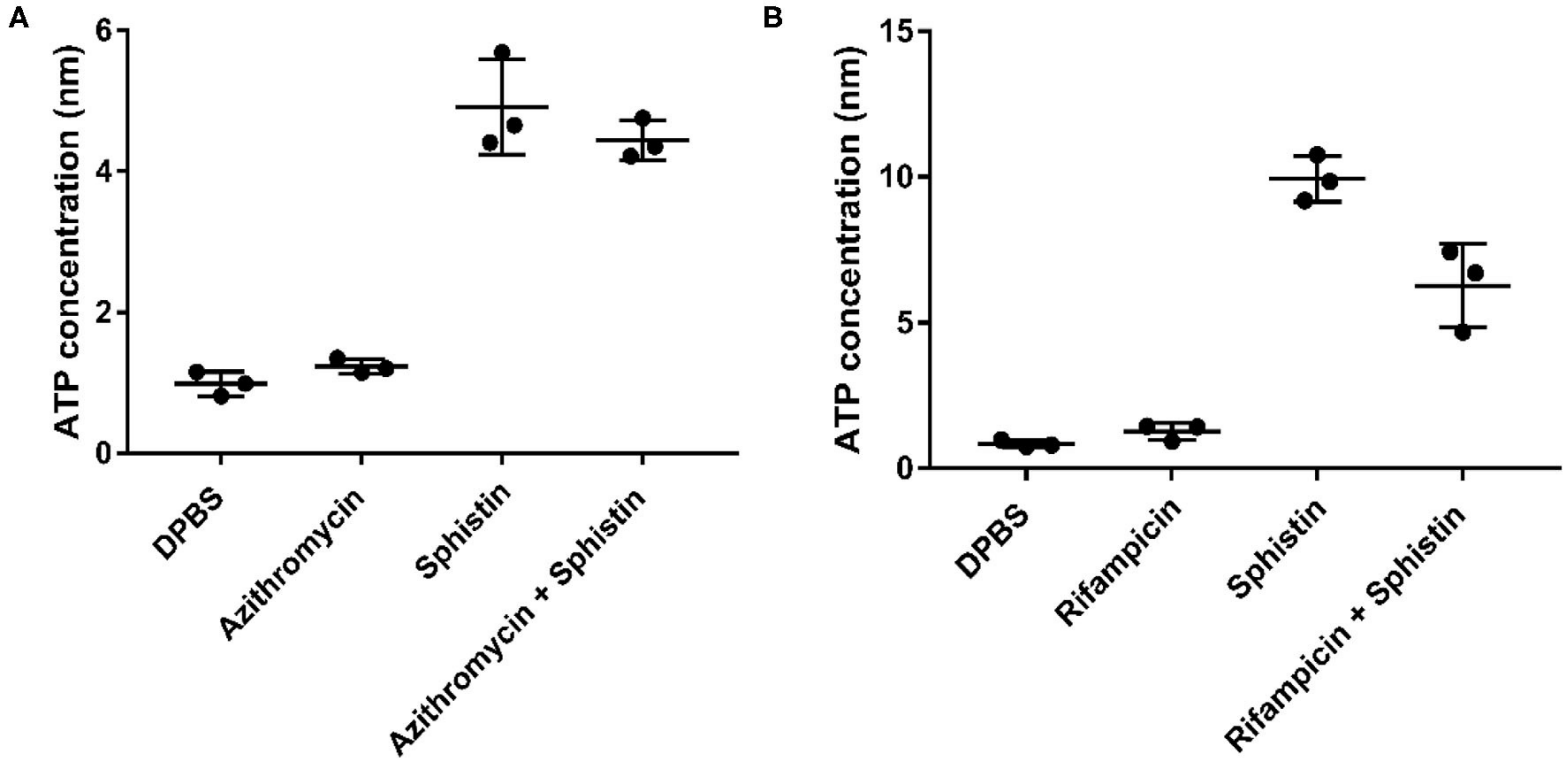
Extracellular ATP release in different treatment groups after exposure of *P. aeruginosa* to **(A)** DPBS, Sphistin (6 μmol·L^−1^), and/or azithromycin (18 μg·ml^−1^) for 2 h; **(B)** DPBS, Sphistin (1.5 μmol·L^−1^), and/or rifampicin (0.625 μg·ml^−1^) for 4 h. The bacteria cell membrane damage induced by the antibacterial agents is accompanied by leakage of intracellular content due to compromised membrane integrity.

**Figure 5:**
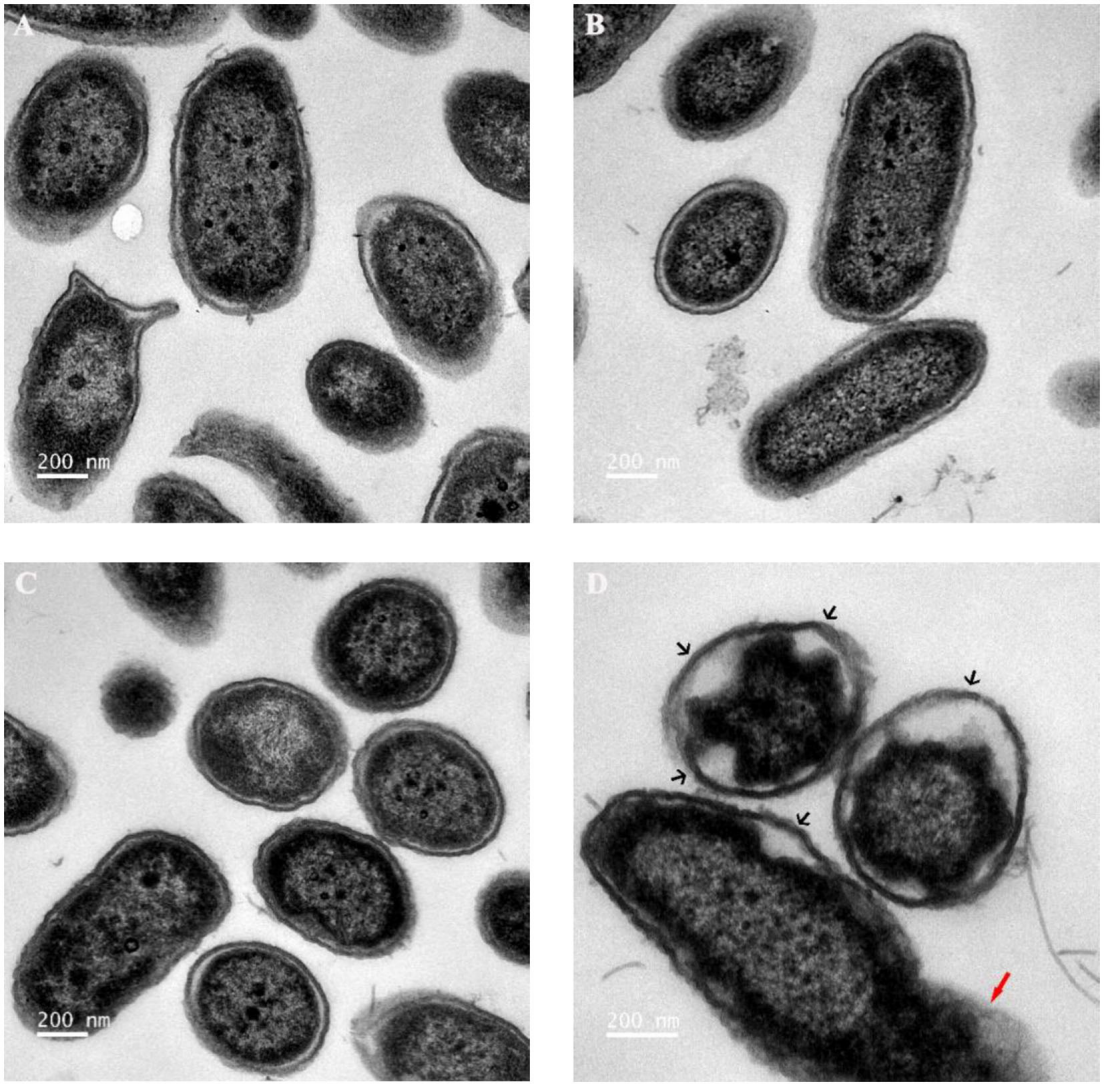
Transmission electron microscopy (TEM) images of *P. aeruginosa* treated with **(A)** DPBS, **(B)** 6 μmol·L^−1^ Sphistin, **(C)** 18 μg·ml^−1^ azithromycin, and **(D)** a combination of 6 μmol·L^−1^ Sphistin and 18 μg·ml^−1^ azithromycin for 1 h at 37°C.

As reported, when the cell membrane was compromised, the barrier function of the cell membrane will be impaired, resulting in the leakage of critical cellular contents (Khara et al., 2015). To further investigate the mechanism of the combination of Sphistin with rifampicin and azithromycin, we evaluated the changes in membrane permeability by measuring the extracellular ATP levels after the two combination group treatments. Compared with the DPBS group, extracellular ATP could not be detected when both the antibiotics were incubated with *P. aeruginos*a. However, bacteria treated with Sphistin alone or in combination with the two tested antibiotics can induce the release of ATP from bacterial cells (Figures 4A,B). Moreover, the leakage of intracellular ATP would not increase, regardless of Sphistin in combination with the antibiotics or used alone. Meanwhile, the leakage of intracellular ATP levels would increase with the extension of time, even at lower concentration of Sphistin. In addition, we also used TEM to observe changes in morphology of *P. aeruginosa* cells or cell membranes. After treatment with DPBS, the control cells had a complete cell morphology (Figure 5A), and no significant changes were observed when the bacterial cells were incubated with Sphistin or azithromycin (Figures 5B,C). The bacteria were then treated with a combination of Sphistin with azithromycin. After 1 h, we observed a significant separation of the cell membrane and cytoplasm in the bacterial cells (Figure 5D, black arrowheads), and remarkable leakage of cellular contents also appeared at the same time (Figure 5D, red arrowheads).

### Sph_12−38_ in Combination With Either Rifampicin or Azithromycin to Promote the Wound Healing

**Figure 6:**
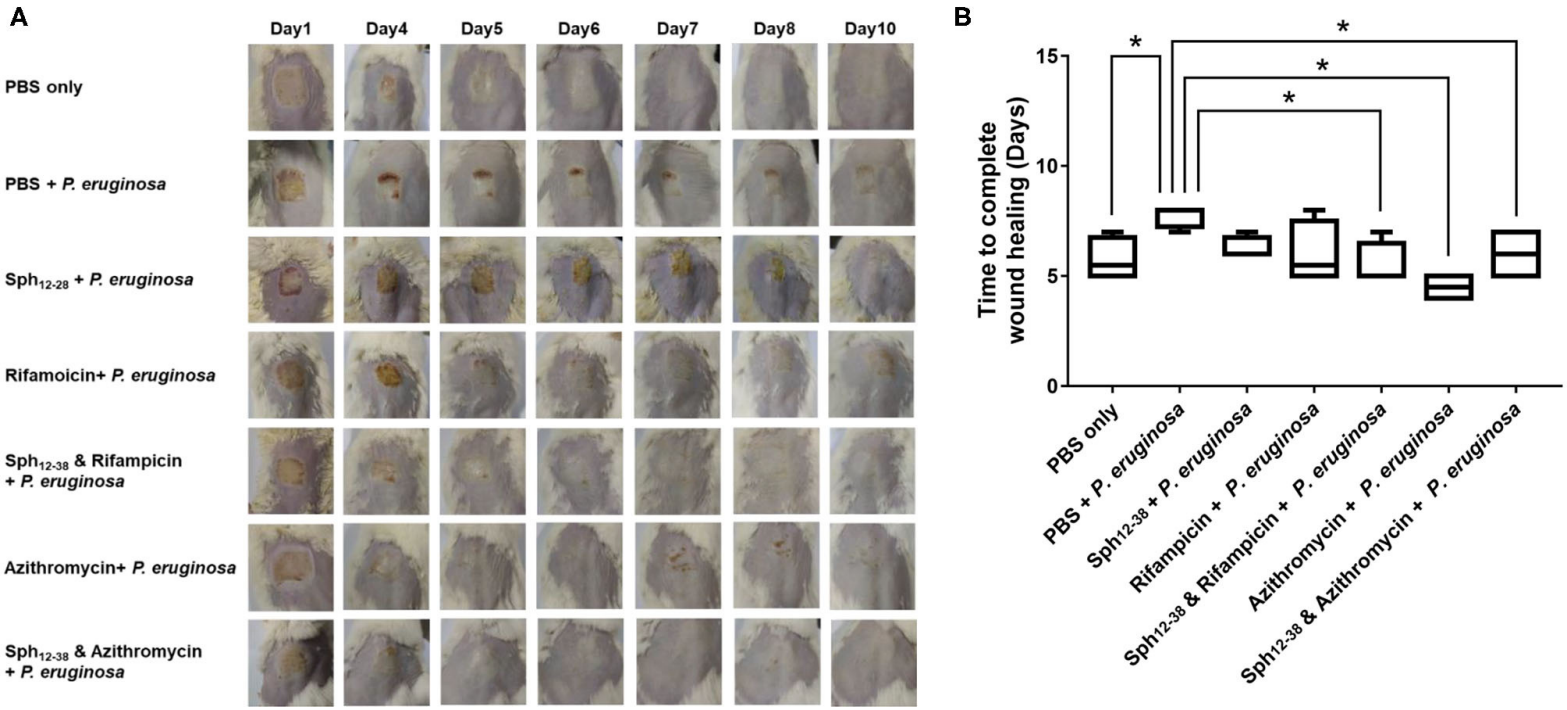
Effect of Sph_12−38_ in combination with rifampin and azithromycin, respectively, on wound healing *in vivo*. **(A)** Insertion status of the wound areas on days 1, 4, 5, 6, 7, 8, and 10 after injury. **(B)** Time to complete wound healing in each group. Data are presented as the means ± standard deviations (*n* = 6 mice per group). *P < 0.05 for Sph_12−38_ in combination with rifampicin-treated mice, Sph_12−38_ alone, and in combination with azithromycin-treated mice, respectively, vs. PBS-treated mice. Differences among groups were evaluated by using Bonferroni correction of variance.

A mouse wound model was used to test the antibacterial activity of Sph_12−38_ in combination with rifampicin and azithromycin *in vivo*. On the dorsal part of each mouse, the epidermis was damaged with medical pressure-sensitive adhesive tape and then the dermis was exposed. Afterwards, 10^8^ CFU of the *P. aeruginosa* were evenly smeared onto the wound areas (Figure 6A). When the wound areas were not infected with *P. aeruginosa*, only the phosphate-buffered saline (PBS) was injected, and all wounds healed within 5–7 days (Figures 6A,B). Meanwhile, for mice infected with *P. aeruginosa*, PBS, Sph_12−38_, rifampicin, azithromycin, and Sph_12−38_ in combination with rifampicin or azithromycin were injected into the wound skin, respectively. When the infected mice were only inoculated with PBS, the wound healing process became slower and was not completed until 7–8 days postinjection. Compared with the PBS group, the injection of Sph_12−38_ or rifampicin alone could not significantly shorten the healing time. In contrast, when the infected mice were injected with Sph_12−38_ in combination with rifampicin, the wound could be completely recovered within 5 to 7 days (*P* < 0.05). As for azithromycin, when used alone or in combination with Sph_12−38_, its efficacy seemed to be even better than the combination of Sph_12−38_ and rifampicin, and the wound completely healed within only 4–5 days (Figures 6A,B).

## Discussion

Since penicillin was introduced into clinical treatment in the 1940s, antibiotics abuse has never ended throughout the past 70 years. The abuse of antibiotics, including overuse and misuse of antibiotics, and the limited availability of new antibiotics have caused the global antibiotic resistance crisis (Ventola, 2015). The abuse of antibiotic inevitably leads to the rapid emergence of drug-resistant bacteria including multidrug resistant (MDR) bacteria, and even the extremely drug-resistant (XDR) or totally drug-resistant (TDR) phenotypes worldwide, which have seriously endangered the efficacy of antibiotics and have become a serious threat to human health (French G. L., 2010; Magiorakos et al., 2012). Although it is known that the resistance determinants are already presented in the microorganisms prior to the introduction of antibiotics and most of them are found in natural antibiotic-producing microorganisms (Levy, 1992), the intensive use of antibiotics indeed has dramatically increased the frequency of resistance among nearly all pathogens that greatly weaken therapeutic options and the medical advantages in the post-antibiotic era that had almost been lost to date (Guay, 2008; Lew et al., 2008; Woodford and Livermore, 2009). Therefore, reducing the use of antibiotics had been proposed, through which the selection pressure for acquired resistance will be reduced and the antibiotic-sensitive bacteria will be recovered, enabling them to eventually defeat resistance strains over time (Levin et al., 1997; Andersson and Levin, 1999). AMPs with a membrane targeting effect provide possibilities to use AMPs in combination with multiple antibiotics for treatment of pathogens, thereby enhancing the efficacy of those antibiotics (Cassone and Otvos, 2010; Haney et al., 2017). Among drug-resistant bacteria, *P. aeruginosa* is one of the most common hospital-acquired and nosocomial conditioned pathogens. It is much prone to acquire multidrug resistance; e.g., some strains of MDR *P. aeruginosa* have been found to be resistant to almost all antibiotics, including aminoglycosides, cephalosporins, fluoroquinolones, and carbapenems (C. D. C. P. (US) Centres for Disease Control and Prevention (US)., 2013; Frieden, 2013). Therefore, it is necessary to explore new agents that could be substituted for antibiotics.

Rifampicin is a semisynthetic antibiotic derived from rifamycin, and it was introduced as an effective medicine to treat tuberculosis; the primary efficacy of rifampicin was against Gram-positive bacteria (Bliziotis et al., 2007). Several studies have revealed that rifampicin in combination with colistin/polymyxins *in vitro* (Giamarellos-Bourboulis et al., 2003; Tascini et al., 2004; Yang et al., 2016) and *in vivo* (Cirioni et al., 2007; Cai et al., 2018) showed effective antibacterial activity against MDR *P. aeruginosa*. For example, four patients infected with sepsis or pneumonia caused by MDR *P. aeruginosa* were successfully cured with the addition of rifampicin to colistin (Tascini et al., 2004). The colistin/polymyxins have an amphipathic structure with clusters of hydrophobic and positively charged regions, and this structural property seems to be closely related to their antibacterial activity (Wade et al., 1990; Scott et al., 1999). In fact, the amphipathic structure with hydrophobic and positive charge is also the classical structural feature of the cationic AMPs; therefore, the combination of rifampicin and two α-helical cationic AMPs, magainin II and cecropin A, also showed synergistic antibacterial effect against *P. aeruginosa* strains *in vitro* and *in vivo* (Cirioni et al., 2008). In addition to rifampicin, it has been proven that the combination of colistin and azithromycin showed synergistic and additive activity against the Gram-negative bacteria *Acinetobacter baumannii* and *P. aeruginosa*, respectively (Timurkaynak et al., 2006). Two AMPs, Sphistin with 38 aa that is derived from the N-terminal of histone H2A in *S. paramamosain* and the truncated short fragment Sph_12−38_, both have potent *in vitro* antibacterial activity against several Gram-positive and Gram-negative bacteria and some fungi. Both Sphistin and Sph_12−38_ showed typical features of cationic AMPs, including amphiphilic α-helical second structure and positive charge net. Therefore, both of these two cationic AMPs were used in combination with rifampicin and azithromycin to treat *P. aeruginosa* in this study.

Outer membranes of Gram-negative bacteria are formed by a divalent cation-crosslinked matrix of lipopolysaccharide (LPS) molecules on the outer leaflet, and via displacing the LPS-bound metals, they could be disrupted by a diverse structural class of polycations (Vaara, 1993; Livermore, 2002; Schuldiner, 2006; Tenover, 2006). It has been proven that polymyxins could bind the lipid A moiety of LPS and perturb the bacterial cell membranes (Evans et al., 1999). Similar to the polymyxins, the cationic AMPs mainly targeted the bacterial cell membranes, and the common features shared by these peptides are that they are prone to form amphipathic structures and then cluster the basic and hydrophobic amino acids into specific regions (Vaara and Porro, 1996; Yeaman and Yount, 2003). Therefore, the cationic AMPs could bind the LPS by electrostatic and hydrophobic interactions and have antibacterial activity (Iwagaki et al., 2000; Yethon and Whitfield, 2001). Meanwhile, the antimicrobial mechanism of Sphistin was suggested to have the capability of attaching the cell membrane and permeabilizing the bacterial cell membranes to kill the pathogens (Chen et al., 2015). Therefore, when *P. aeruginosa* was treated with the combination of Sphistin and two antibiotics, the permeabilization of the bacterial cell membranes facilitated the uptake of the two antibiotics; rifampicin would bind to and inhibit the bacterial DNA-dependent RNA polymerase (Campbell et al., 2001), and azithromycin would inhibit RNA-dependent protein synthesis of the bacteria (Mazzei et al., 1993; Alvarez-Elcoro and Enzler, 1999); finally, both antibiotics would induce cell death. Just as Figure 4 showed, there was no difference in the leakage of the intracellular ATP between Sphistin used alone or the combination of Sphistin with two antibiotics. Indeed, these results further verified that even in the synergy system, only Sphistin induced the permeabilization of the bacterial cell membranes. Taken together, our results agreed with previous studies (Cirioni et al., 2008) that when the AMPs are combined with the antibiotics to treat the pathogens, the AMPs induced the permeabilization of the pathogen cell membranes and subsequently promoted uptake of the antibiotics, allowing the antibiotics to interact with their intracellular targets easily and finally kill the pathogens.

Among the eight selected antibiotics, the two antibiotics azithromycin and rifampicin in combination with the AMPs Sphistin or Sph_12−38_ showed a synergistic effect against *P. aeruginosa*. However, the other six antibiotics had no significant synergistic effect against *P. aeruginosa* when each of them was combined with the AMPs. Among the six antibiotics, as previously reported (Menninger and Otto, 1982; Nord and Kager, 1983; Gardner and Hill, 2001; Raether and Hanel, 2003; Tasca et al., 2003), tinidazole and clindamycin also have intracellular targets. Among the remaining four antibiotics, the three antibiotics including penicillin, ceftizoxime, and cefotiam are all β-lactam antibiotics, while vancomycin is a glycopeptide antibiotic (Kahne et al., 2005). All of these four antibiotics can inhibit the synthesis of bacteria cell walls as previously reported (Wise and Park, 1965; Waxman et al., 1980; Kahne et al., 2005).

Tinidazole is a structural analog of metronidazole. Both tinidazole and metronidazole are active against anaerobic organisms or protozoa. The antibacterial mechanism is briefly described as follows. When tinidazole diffused into bacterial cells, the nitro group of tinidazole will be reduced to short-lived and toxic free radicals. The toxic intermediates covalently bind to DNA, causing DNA damage and ultimately cell death (Nord and Kager, 1983; Gardner and Hill, 2001; Raether and Hanel, 2003; Tasca et al., 2003). With the decrease of intracellular concentration of tinidazole due to the reduction reaction, more tinidazole could enter the cells, thereby maintaining the inhibition activity of anaerobic bacteria. In aerobic bacteria and mammalian cells, they have relatively high redox potentials and are also rich in oxygen molecules than anaerobic organisms or protozoa, which will hinder the reduction reaction (Nord and Kager, 1983; Tasca et al., 2003). Because *P. aeruginosa* is an aerobic bacterium, we speculated that tinidazole will not have much effect on *P. aeruginosa* due to the lack of anaerobic condition in cells. Therefore, even if tinidazole is used in combination with either Sphistin or Sph_12−38_, more tinidazole might get access into the bacterial cells, but tinidazole cannot or rarely show effective bactericidal activity against *P. aeruginosa*. Meanwhile, the concentration of the AMPs in the synergistic system was only 1/2 MIC, which cannot inhibit *P. aeruginosa* alone as testified in the study. Generally, only when the concentration of the AMPs was equal to or greater than the MIC could the AMPs effectively inhibit the target bacteria. Thus, although when in combination with the concentration of 1/2 MIC, Sphistin or Sph_12−38_ can also accelerate entrance of tinidazole into *P. aeruginosa*, it still did not produce a significant synergistic effect.

Another antibiotic with intracellular action, clindamycin, belongs to lincosamide, which is a 50S ribosome inhibitor. It inhibits bacteria by preventing peptidyl-tRNAs from entering the ribosome and finally triggers the dissociation of the peptidyl-tRNA (Menninger and Otto, 1982). As reported in previous studies, in general, aerobic Gram-negative (G–) bacteria are resistant to clindamycin, but clindamycin is effective against the Gram-positive (G+) bacteria, such as *S. aureus, Streptococcus pyogenes, S. pneumoniae*, and *Streptococcus viridans*. The synergistic effect of clindamycin combined with AMPs has been reported previously (Spizek et al., 2004; Nguschwemlein et al., 2014; Chernysh et al., 2018). For the G+ bacteria, when clindamycin was used in combination with the AMPs cyclooctapeptides (CPs, including CPs 1–3, 5–7, 10, 11) against *S. aureus*, all combinations showed partial synergistic effects (0.5 ≤ FICI < 1) (Nguschwemlein et al., 2014). When clindamycin was used in combination with FLIP7, the AMP complex from the blowfly *Calliphora vicina* contains a combination of defensins, cecropins, diptericins, and proline-rich peptides against *S. aureus*, showing a synergistic effect (Chernysh et al., 2018). To our knowledge, for the G– bacteria, only one literature reported that clindamycin combined with the peptidomimetic 26 against *K. pneumoniae* ST258 showed a synergistic effect (Baker et al., 2019); however, no any related studies on the combined use of clindamycin and AMPs against *P. aeruginosa* have been reported. There are three main types of bacterial resistance mechanisms to clindamycin as reported, including MLS_b_ resistance, mutations in ribosome binding sites, and active efflux of antibiotics from the periplasmic space (Spizek et al., 2004), which mainly occurs in Gram-negative bacteria (Leclercq and Courvalin, 1991). As mentioned in the *Introduction*, the transmembrane efflux pumps are also considered be the cause of the intrinsic resistance of *P. aeruginosa*, through which the antibiotics can be effectively taken out of the bacteria (Li et al., 1994). The concentration of clindamycin used in the study was 25 μg·ml^−1^, which is much lower than the MIC of clindamycin against *P. aeruginosa* (1,000 μg·ml^−1^). At the same time, in the synergistic system, only 1/2 MIC of the AMPs were used. At this concentration, the membrane of *P. aeruginosa* might not be completely disrupted, so the transmembrane efflux pumps of *P. aeruginosa* might still have a certain effect, and the intracellular concentration of the antibiotics might be reduced by the transmembrane efflux pumps of *P. aeruginosa*. As a result, the intracellular clindamycin cannot effectively inhibit the synthesis of bacterial protein. Therefore, the combination of clindamycin and the AMPs had no obvious synergistic effect on *P. aeruginosa*.

In the clinic, penicillin, ceftizoxime, cefotiam, and vancomycin are usually used to treat Gram-positive bacterial infections. Penicillin, ceftizoxime, and cefotiam are all β-lactam antibiotics. They can inhibit peptide bond formation by competitively binding penicillin binding proteins (PBP), prevent the cross-linking of peptidoglycan units, and inhibit the synthesis of bacteria cell walls (Wise and Park, 1965; Waxman et al., 1980). In addition, vancomycin is a glycopeptide antibiotic that can specifically bind to D-Ala-D-Ala dipeptide of the peptidoglycan intermediates, inhibit transglycosylation and/or transpeptidation, overall weaken the peptidoglycan layers, and make the bacterial cells susceptible to changes in osmotic pressure, sequentially inducing cell lysis (Kahne et al., 2005). For the bacterial cell walls, there are three main layers on the cell walls of Gram-negative bacteria, including the outer membrane (OM), the peptidoglycan cell wall, and the cytoplasmic or inner membrane (IM) (Glauert and Thornley, 1969). The outer membrane is mainly composed of LPS (Kamio and Nikaido, 1976), which can protect Gram-negative bacteria from environmental influences by excluding toxic molecules and providing an extra stable layer around the cell. Compared with Gram-positive bacteria, the peptidoglycan layer of Gram-negative bacterial cells is relatively thin. The peptidoglycan layer in the cell walls of *P. aeruginosa* is only 2.41 ± 0.54 nm thick (Matias et al., 2003), while the Gram-positive bacteria lack the outer membrane and are surrounded by the peptidoglycan layers that are several times thicker than Gram-negative bacteria. The thickness of those peptidoglycan layers ranges from 30 to 100 nm (Silhavy et al., 2010).

For the Gram-negative bacteria *P. aeruginosa*, the outer membrane would act as a potential barrier to the entrance of antibiotics. In the study, when the antibiotics were used in combination with AMPs Sphistin or Sph_12−38_, the membrane perturbations caused by the AMPs might allow more antibiotics to enter the bacterial cells. Nevertheless, since the main target for the β-lactam antibiotics (like three antibiotics in the study) and vancomycin is peptidoglycan synthesis, even if the membrane perturbations accelerated the entry of these four antibiotics into bacteria and possibly affect the peptidoglycan synthesis of *P*. *aeruginosa*, their action could not affect the integrity of the outer membrane. In addition, a low concentration of the AMPs (<1/2 MIC) could not completely destroy the structure of the outer membrane, and the cell morphology of *P*. *aeruginosa* can maintain relative integrity, indicating that the bacteria remained alive. Therefore, when these two antibiotics were used in combination with the AMPs against *P*. *aeruginosa*, no significant synergistic effect was observed.

The *in vitro* antibacterial tests indicated that the combination of Sphistin with rifampicin and azithromycin killed the pathogens efficiently. To demonstrate the synergy effects further, we tested the antibacterial efficiency *in vivo*. Similar to the experimental results *in vitro*, the remarkable effect appeared using Sph_12−38_ in combination with rifampicin that promoted the wound healing significantly (Figures 6A,B), whereas no significant effect was found using Sph_12−38_ or rifampicin alone. The underlying mechanism was presumed as follows. The AMPs could induce the permeabilization of bacterial cells, facilitating rifampicin to access the cells and bind their binding sites; alternatively, the peptides stimulate the immune systems of the host and then rifampicin could play an antibacterial role independently of the AMPs (Vaara and Porro, 1996; Yeaman and Yount, 2003; Balakrishna et al., 2006). Nevertheless, unlike rifampicin, azithromycin alone or in combination with Sph_12−38_ significantly facilitated the wound healing. Otherwise, Sphistin/Sph_12−38_ could bind to LPS and permeabilize the bacterial membrane; when combined with rifampicin and azithromycin, Sphistin/Sph_12−38_ promoted the intracellular uptake of the antibiotics and subsequently enhanced the bactericidal activity of both agents against *P. aeruginosa*. Although *P. aeruginosa* was non-susceptible to rifampicin or azithromycin, when in combination with Sphistin/Sph_12−38_, they all showed higher antibacterial efficiency; the combination of Sphistin/Sph_12−38_ with rifampicin and azithromycin might be potentially used for the prevention and treatment of infections caused by *P. aeruginosa*; however, more work needs to be done in the future.

## Data Availability Statement

The raw data supporting the conclusions of this article will be made available by the authors, without undue reservation.

## Ethics Statement

The animal study was reviewed and approved by Xiamen University Laboratory Animal Management and Ethics Committee.

## Author Contributions

K-JW design the research work, supervised, and revised the manuscript. JL performed the experiment, co-designed, and wrote the paper. XW and HZ participated the antibacterial experiment and the transmission electron microscopy (TEM) experiment, and verified the overall replication/reproducibility of results/experiments and other research outputs. HP provided the antimicrobial peptides and co-designed the *in vivo* experiment. K-JW and FC supervised and revised the research work. All authors contributed to the article and approved the submitted version.

## Conflict of Interest

The authors declare that the research was conducted in the absence of any commercial or financial relationships that could be construed as a potential conflict of interest.
